# Endovascular Hepatic Artery Stents in the Modern Management of Postpancreatectomy Hemorrhage

**DOI:** 10.1097/AS9.0000000000000038

**Published:** 2021-02-08

**Authors:** Louise M. Finch, Minas Baltatzis, Sam Byott, Anantha-Krishnan Ganapathy, Nirmal Kakani, Edward Lake, Rosemary Cadwallader, Can Hazar, Dare Seriki, Stephen Butterfield, Santhalingam Jegatheeswaran, Saurabh Jamdar, Nicola de Liguori Carino, Ajith K. Siriwardena

**Affiliations:** From the * Regional Hepato-Pancreato-Biliary Surgery Unit, Manchester Royal Infirmary, Manchester, United Kingdom; † Vascular Radiology Department, Manchester Royal Infirmary, Manchester, United Kingdom; ‡ Faculty of Biology, Medicine and Health, University of Manchester, Manchester, United Kingdom.

**Keywords:** endovascular stent, hepatic artery, postpancreatectomy hemorrhage

## Abstract

**Background:**

Postoperative hemorrhage is a potentially lethal complication of pancreatoduodenectomy. This study reports on the use of endovascular hepatic artery stents in the management of postpancreatectomy hemorrhage.

**Methods:**

This is a retrospective analysis of a prospectively maintained, consecutive dataset of 440 patients undergoing pancreatoduodenectomy over 68 months. Data are presented on bleeding events and outcomes, and contextualized by the clinical course of the denominator population. International Study Group of Pancreatic Surgery terminology was used to define postpancreatectomy hemorrhage.

**Results:**

Sixty-seven (15%) had postoperative hemorrhage. Fifty (75%) were male and this gender difference was significant (*P* = 0.001; 2 proportions test). Postoperative pancreatic fistulas were more frequent in the postoperative hemorrhage group (*P* = 0.029; 2 proportions test). The median (interquartile range [IQR]) delay between surgery and postoperative hemorrhage was 5 days (2–14 days). Twenty-six (39%) required intervention comprising reoperation alone in 12, embolization alone in 5, and endovascular hepatic artery stent deployment in 5. Four further patients underwent more than 1 intervention with 2 of these having stents. Endovascular stent placement achieved initial hemostasis in 5 of 7 (72%). Follow-up was for a median (IQR) of 199 days (145–400 days) poststent placement. In 2 patients, the stent remained patent at last follow-up. The remaining 5 stents occluded with a median (IQR) period of proven patency of 10 days (8–22 days).

**Conclusions:**

This study shows that in the specific setting of postpancreatoduodenectomy hemorrhage with either a short remnant gastroduodenal artery bleed or a direct bleed from the hepatic artery, where embolization risks occlusion with compromise of liver arterial inflow, endovascular hepatic artery stent is an important hemostatic option but is associated with a high risk of subsequent graft occlusion.

## Abstract 2

Mini-abstract: In a consecutive cohort of 440 undergoing pancreatoduodenectomy, 67 had postpancreatectomy hemorrhage. Where embolization was impossible due either to short gastroduodenal artery remnant or bleed directly from hepatic artery, endovascular stent was deployed with initial hemostasis in 5 (72%). This study reports on endovascular hepatic artery stents in the modern management of postpancreatectomy hemorrhage.

## INTRODUCTION

Postoperative hemorrhage is a potentially lethal complication of pancreatoduodenectomy.^1^ It can be heralded by a sentinel bleed^2^ but can also occur suddenly and without prior warning. As postpancreatectomy hemorrhage is an infrequent complication, evidence for management accrues from case series.^3–5^

Treatment of postpancreatectomy hemorrhage is difficult. Early postoperative hemorrhage is typically managed by reoperation.^6^ Later postoperative hemorrhage is often associated with disruption of the pancreatoenteric anastomosis, perianastomotic abscess, and pancreatic fistula.^7,8^ Perivascular sepsis leads to pseudoaneurysm formation, most frequently at the site of division of the gastroduodenal artery (GDA) or along the course of the hepatic artery.^9^ In addition, visceral artery pseudoaneurysms can arise along the course of any of the branches of the celiac axis or superior mesenteric artery. In modern practice, computed tomographic (CT) angiography is typically the first diagnostic test of choice in patients with late postoperative hemorrhage who are sufficiently stable from a cardiovascular perspective to tolerate this.^10^

In patients with positive findings of extravasation or pseudoaneurysm on CT angiography and who continue to be sufficiently stable to permit interventional radiological treatment, selective mesenteric angiography is the next step.^11,12^

Once arterial extravasation has been confirmed, angiographic hemostatic techniques are the preferred treatment in this setting but are technically highly challenging. Placement of coils or liquid embolics to exclude a pseudoaneurysm or occlude the bleeding vessel are accepted techniques, but in the specific setting of postpancreatoduodenectomy hemorrhage with a short course of the remnant GDA or a direct bleed from the hepatic artery, these techniques may not be feasible. As a result, interest has focused on the use of endovascular stents to occlude the GDA origin and also to exclude bleeding points along the course of the common hepatic artery.^13^ However, in addition to the risks of injury to vasculature during stent deployment, there are also risks of rebleeding and stent thrombosis with resultant hepatic injury.^13,14^ The outcomes of endovascular hepatic artery stenting after pancreatoduodenectomy are also relatively unknown.^15^ This contemporary series describes the presentation and management of postpancreatoduodenectomy hemorrhage and seeks to integrate the use of endovascular hepatic artery stents into a modern management algorithm.

## METHODS

### Design

This is a retrospective analysis of a prospectively maintained dataset of a clinical cohort of patients undergoing pancreatoduodenectomy. The study evaluates the diagnosis and management of postpancreatoduodenectomy hemorrhage with reference to the use of endovascular hepatic artery stents.

### Setting

This is a single-center study undertaken in the regional hepato-pancreato-biliary service of the Manchester Royal Infirmary, Manchester, United Kingdom. This hospital also houses a regional vascular and endovascular service with round-the-clock interventional vascular radiology cover and access to an integrated vascular/radiology hybrid suite.

### Patients

The prospectively maintained database of pancreatic resections was interrogated for the period October 1, 2014, to June 1, 2020. All patients who underwent pancreatoduodenectomy during this time period constitute the study population. All perioperative complications were prospectively recorded in an institutional database and assessed weekly as part of the unit’s morbidity and mortality review program. Data on demographic details, operative procedure, postoperative course, and information on final histologic diagnosis were collected. Clinical evidence of bleeding was considered when a patient met 1 or more of the following criteria: hemodynamic instability, drop of hemoglobin ≥ 2 g/dL within a 24-hour time period, fresh blood in abdominal drain, and/or hematemesis or melena. Diagnosis and management of the postoperative bleed, including use of CT angiography and other interventions (reoperation, endoscopic intervention, mesenteric angiography, and stenting or embolization), were recorded. Liver function tests (LFTs), including alanine aminotransferase (ALT), alkaline phosphatase, and bilirubin, were recorded from prior to stent insertion to 14 days poststent procedure. Duration of hospital stay was recorded. Episode-related mortality was defined as death after pancreatoduodenectomy up to 90 days after surgery. In addition, patients who underwent hepatic artery stent placement were followed-up from time of deployment until data analysis.

### Relevant Aspects of Surgical Technique

Pancreatoduodenectomy was performed with either a pylorus-preserving modification or a classic resection according to individual surgeon preference. Eight patients underwent laparoscopic pancreatoduodenectomy. All others underwent surgery via the open approach. Restoration of pancreatic-enteric continuity was by pancreatojejunostomy in all cases. Portal/superior mesenteric vein resection and reconstruction were performed when indicated. Reconstruction after vein resection was mainly by primary venous anastomosis. Two patients underwent graft reconstruction of the portal vein. These 2 patients had no postoperative bleeding events. All patients had postoperative surgical drains using a closed nonsuction system (Portex Robinson Drainage System; Smiths Medical International Ltd, Watford, United Kingdom). Drain fluid content was routinely analyzed for amylase on the 3rd postoperative day. Drains were maintained until resolution of output if the amylase content was greater than 3 times the upper limit of the laboratory normal serum value. Unit policy recommended that patients were routinely prescribed postoperative subcutaneous octreotide. Low molecular weight heparin was prescribed after confirmation of normal postoperative blood clotting profile.

### Relevant Aspects of Endovascular Stent Placement

Selective mesenteric angiography was performed via a common femoral artery approach. If the source of active bleeding was a nonessential branch or a vessel that could be sacrificed, then coil or liquid embolization was performed to achieve hemostasis. If the bleeding point was from a short GDA remnant or from the main hepatic artery and thus not amenable to embolization without risk of compromise to liver inflow, an hepatic artery stent was deployed. Six patients had balloon-expandable stent placement using Bentley BeGraft (BeGraft; Bentley, Hechingen, Germany) or Jotec E-ventus (Jotec, Hechingen, Germany) stents. One patient had a self-expanding Gore Viabahn stent (WL Gore and Associates, Flagstaff, AZ). Stent grafts were sized within 1 mm diameter of the target vessel. Completion angiography demonstrated an appropriately sited, patent stent graft with angiographic cessation of bleeding in all cases. Antiplatelet therapy was not routinely used after endovascular hepatic artery stent placement.

### Definitions of Postoperative Complications

Postpancreatectomy hemorrhage was defined as described by the International Study Group of Pancreatic Surgery.^16^ Postoperative pancreatic fistula was also defined according to the updated International Study Group of Pancreatic Surgery definition.^17^

### Data Extraction and Recording

Data were extracted on clinical course and outcome by 2 coauthors (L.M.F., M.B.) and used to populate a bespoke database for analysis. Data capture was confirmed by cross-checking of procedure records and complications databases. A further check was introduced by cross-checking with angiography suite records. CT scans were regarded in 2 categories: scans undertaken for assessment of nonhemorrhagic postoperative complications and CT scans undertaken (to CT angiography protocols) for assessment of suspected postoperative hemorrhage. If any patient undergoing CT for suspected nonhemorrhagic complications was thought to show evidence of bleeding, this individual was included in the postoperative hemorrhage dataset. Data were then used to populate bespoke tables on procedure and outcome together with a detailed individual-patient report on those who underwent endovascular stent.

### Statistical Analysis

Statistical analysis was performed with MiniTab statistical software (MiniTab LLC, State College, PA) and StatsDirect statistical software (StatsDirect Ltd, Merseyside, United Kingdom). Scale variables were expressed as median ± range and categorical parameters as absolute count and percentage. The Anderson-Darling test was applied to determine data normality. Where data were categorical, they were compared using the 2 proportions test and where continuous a 2-sample *t* test was used for normal data and a binary logistic regression for non-normal data. One-way ANOVA was used to analyze differences in enzymatic LFTs over time. Statistical significance was accepted at the *P* < 0.05 level. Graphs were produced using GraphPad Prism 6 (GraphPad Software, San Diego, CA).

### Ethics Approval

The study was regarded as an audit by the Hospital Trust’s Research and development team and registered with the audit department (audit number 7132; March 7, 2017).

## RESULTS

### Population of Patients Undergoing Pancreatoduodenectomy (n = 440)

During the study period, 440 patients underwent pancreatoduodenectomy, of whom 67 (15%) had a postoperative hemorrhage. Demographic and operative details of the patient population are seen in Table 1. Overall, 106 (24%) had a biochemical pancreatic fistula after resection. Octreotide was administered to 258 (58%).

**TABLE 1.** Details of Patients Undergoing Pancreatoduodenectomy (n = 440)

	Patients With Postoperative Hemorrhage, N = 67	Patients Without Postoperative Hemorrhage, N = 373	*P*
Age (years), median (IQR)	67 (59–72)	66 (59–73)	0.980
Gender			
Male	50 (75)	203 (54)	0.001*
Female	17 (25)	170 (46)	
Type of surgery			
Classic Whipple	37 (55)	219 (59)	0.509
PPPD	25 (37)	145 (39)	0.788
Total pancreatectomy	5 (8)	9 (2)	0.029*
Postoperative pancreatic fistula	34 (51)	123 (33)	0.006*
Biochemical leak	24 (36)	106 (28)	0.196
Type B	6 (9)	15 (4)	0.069
Type C	4 (6)	2 (1)	0.003*
Use of octreotide	51 (76)	258 (69)	0.251
Onset (days), median (IQR)	5 (2–14)		
Early ≤ 24 hours	15 (22)		
Late > 24 hours	52 (78)		
Presentation of bleed			
Blood in drain	30 (45)		
Hematemesis/melena	19 (28)		
Cardiovascular instability	9 (14)		
Hemoglobin drop	7 (10)		
Bleeding at wound site	2 (3)		
CT angiography	39 (58)		
Arterial extravasation on CT angiography	6 (9)		
Required intervention†	26 (39)		
Reoperation	15 (22)		
Embolization	9 (13)		
Stenting	7 (10)		
Mortality			
90 days after surgery	8 (12)	10 (3)	0.001*

Figures in parentheses represent percentages unless otherwise stated.

*Statistical significance (*P* < 0.05).

†Intervention was undertaken in 26 patients: 12 underwent reoperation alone, 5 embolization alone, and 5 underwent endovascular stenting of the common hepatic artery alone. Four further patients underwent more than 1 intervention as follows: 2 underwent reoperation followed by embolization, 1 underwent stent followed separately by embolization, and 1 underwent reoperation, endovascular stent, and then total (completion) pancreatectomy.

PPPD indicates pylorus-preserving pancreatoduodenectomy.

### Characteristics of Patients Experiencing Postpancreatectomy Hemorrhage (n = 67)

Fifty (75%) of patients were male and this gender difference was significant (*P* = 0.001; 2 proportions test). There was no difference between patients who had postoperative hemorrhage and those who did not in terms of age, type of pancreatoduodenectomy, vein resection, or laparoscopic/open approaches (Table 1). Postoperative pancreatic fistulas were seen more frequently in the postoperative hemorrhage group (*P* = 0.006; 2 proportions test). Although 51 (76%) of the postoperative hemorrhage group received postoperative octreotide, this difference was not significant compared with the denominator population (*P* = 0.251; 2 proportions test).

### Presentation of Postpancreatectomy Hemorrhage (n = 67)

The median (interquartile range [IQR]) delay between surgery and postoperative hemorrhage was 5 days (2–14 days) (Table 1). Fifteen patients had early postoperative hemorrhage within 24 hours of surgery. The mode of presentation is seen in Table 1 with blood in drains being the most common presentation in 30 (45%). CT angiography was undertaken in 39 (58%) with 6 patients showing evidence of arterial contrast extravasation.

### Management of Postpancreatectomy Hemorrhage (n = 67)

Twenty-six (39% of postpancreatectomy hemorrhage) required intervention (Table 1). The remaining 41 were managed conservatively.

**FIGURE 1.**
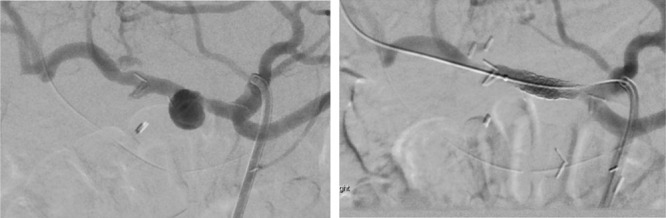
Successful stenting of the hepatic artery to exclude a pseudoaneurysm. A, Angiography demonstrates a 14 mm pseudoaneurysm of the common hepatic artery proximal to the GDA stump. B, 7 x 23 mm balloon-expandable Bentley stent deployed (Bentley Innomed GmbH, Hechingen, Germany) and poststent placement film demonstrating no filling of the pseudoaneurysm.

Of those requiring intervention, 12 underwent reoperation alone, 5 embolization alone, and 5 underwent endovascular stenting of the common hepatic artery alone (Table 1). Four further patients underwent more than 1 intervention. In detail, these were 2 patients who underwent reoperation followed by embolization, 1 patient who had stent and embolization, and 1 patient who underwent reoperation, embolization, endovascular stent, and then total (completion) pancreatectomy. Thus, 9 patients underwent embolization and 7 underwent endovascular hepatic artery stent. Figure 1 shows images from successful angiographic stent placement leading to occlusion of a false aneurysm arising from the common hepatic artery.

Of the 7 undergoing endovascular hepatic artery stent (details in Table 2) (5 as solo procedures 2 in addition to other interventions), 3 (43%) were for patients whose bleeding point was from the common hepatic artery, 3 from the stump of the GDA, and in 1 patient the bleeding point could not be confirmed but was thought to be from the common hepatic artery based on the CT angiographic findings. Endovascular hepatic artery stent placement achieved initial hemostasis in 5 (72%). Two had continued bleeding and a further patient had a delayed rebleed at 20 days. Of those with ongoing bleeding, repeat angiography revealed a bleeding point from a right adrenal pseudoaneurysm distinct from the original bleeding point and treated by embolization in 1 patient. In the second patient, repeat selective mesenteric angiography did not demonstrate any pseudoaneurysm or active bleed. The patient with late-onset rebleeding after endovascular stent was managed by reoperation resulting in total (completion) pancreatectomy.

### Hepatic Enzyme Profiles After Endovascular Stent Placement (n = 7)

**FIGURE 2.**
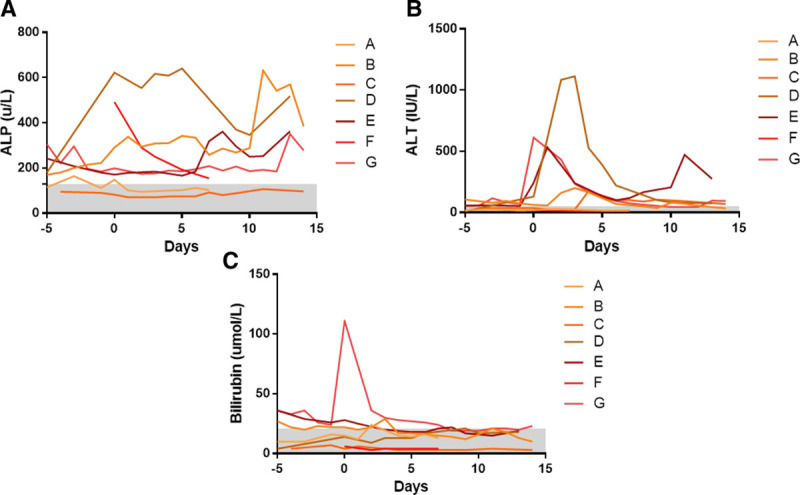
Enzymatic liver function tests before and after hepatic stent placement. A, Alanine aminotransferase, (B) ALP, and (C) bilirubin measured prestent placement and up to 14 days following stent placement. One-way ANOVA test for significance, *P* = 0.623, 0.590, and 0.979, respectively. (Stent placement at day 0 and laboratory normal range of test indicated by gray bar). ALP indicates alkaline phosphatase.

Figure 2 demonstrates that there was elevation of the serum ALT after deployment of endovascular stent. This elevation was not statistically significant (*P* = 0.623; 1-way ANOVA). Serum alkaline phosphatase remained higher than the reference range throughout. Serum bilirubin remained within the reference range, except for 1 large spike in 1 individual at the time of stent insertion. One patient developed ischemic necrosis of the liver following stent occlusion and had deranged LFTs at 30 days poststent insertion (ALT of 145 IU/L, alkaline phosphatase of 713 U/L, and bilirubin 203 μmol/L) and subsequently died.

### Late Sequelae of Endovascular Hepatic Artery Stent (n = 7)

Follow-up was for a median (IQR) of 199 days (145–400 days) poststent placement. In 2 patients, the hepatic artery stent remained patent at last follow-up imaging without any apparent hepatic compromise (598 and 126 days, respectively). The remaining 5 stents occluded with a period of proven median (IQR) patency of 10 days (8–22 days) (time from deployment to scan showing occlusion). In these 5 patients, liver abscess was seen in 3 (43% of cohort) and 1 had patchy multifocal hepatic infarction. Three of these patients died at a median (IQR) 199 days (31–234 days) after stent deployment. Two patients with occluded stents remained alive with no further rebleeding up to the time of analysis (566 and 130 days, respectively).

### Antiplatelet Therapy After Endovascular Hepatic Artery Stent (n = 7)

Three patients (43%) had antiplatelet therapy in the form of either single agent clopidogrel or aspirin. Two of these patients’ stents were patent at the latest imaging with a duration of patency of 680 days and 126 days, respectively.

## DISCUSSION

This cohort study has examined the presentation and management of the potentially lethal complication of postpancreatectomy hemorrhage. The inclusion of the entire cohort of patients undergoing pancreatoduodenectomy in this center during the study period shows that the number of patients requiring intervention for postpancreatectomy hemorrhage is small. The nature of postoperative bleeding and its relative infrequency make randomized evaluation of intervention difficult, and thus, evidence to guide management accrues from case series such as this report. In turn, idiosyncrasies in individual unit policy can produce an element of variation between the practice of this unit and pancreatoduodenectomy on a global perspective. Examples of these include the choice of open/minimally invasive approach, the technique of reconstruction, use of postoperative drains, and type of drain employed. Accepting these limitations, the overall series of 440 consecutive patients undergoing pancreatoduodenectomy in a specialist hepato-pancreato-biliary center is representative of a modern cohort.

Comparing the patients who experienced postpancreatectomy hemorrhage to the denominator cohort, it is interesting that there is a striking gender imbalance with more males experiencing postpancreatectomy hemorrhage. While this could be due to chance or confounding variables such as greater body mass index or proportion of visceral fat, this phenomenon is worthy of further study. Lopes et al^18^ have demonstrated male gender as a risk factor for bleeding after cardiac surgery and hypothesized that the reason could be related to an increased procoagulant tendency in females. Thromboelastographic studies in healthy males and females demonstrate that females have a higher rate of fibrin formation and clot strength.^19^

The next striking difference between the postpancreatectomy hemorrhage group and the denominator population is in the incidence of postpancreatectomy fistula. As expected, the proportion of patients with pancreatic fistula was greater in the hemorrhage group compared with the nonhemorrhage group (*P* = 0.009).^19^

The data presented here can be integrated into a modern management algorithm. Few would argue with a suggestion that early postpancreatectomy hemorrhage should be managed by reoperation.^20^ Definition of the term “early” is clear for patients who bleed within 24 hours of surgery and may also apply to those who bleed in the first 72 hours after resection, but beyond that period, there is arguably an interface zone where early postoperative hemorrhage is progressively replaced by late or reactionary bleeding.

CT angiography is widely used as the first diagnostic test in the setting of late postpancreatectomy hemorrhage. Although the yield in terms of demonstration of arterial extravasation may be low, CT angiography can provide important information on peripancreatic collections, visceral perfusion, etc. Following CT angiography, selective mesenteric angiography is indicated in those patients in whom the scan has demonstrated extravasation or where there is continuing hemorrhage (despite an absence of confirmed extravasation). In both these scenarios, there will be a clinical judgment call to be made between a decision to reoperate or opt for attempted angiographic hemostasis. Fiber-optic endoscopy also has to be considered in the diagnostic options, especially in patients with evidence of luminal bleeding. Endoscopy has the advantage of combining a diagnostic modality with a potential therapeutic option (injection or clipping of a bleeding point).

If a decision is made to undertake selective mesenteric angiography, it is important to include visualization of the celiac axis branches and the superior mesenteric artery. If extravasation from the GDA or the common hepatic artery is confirmed, weight of experience derived from clinical use continues to favor coil or liquid embolization if compromise of flow in the common hepatic artery can be avoided.^21^ However, in settings where the GDA remnant is too short for coil or liquid embolization or where the bleed originates from the main hepatic artery, endovascular stent placement can be considered.

Endovascular hepatic artery stent placement is associated with the specific postprocedure risks of rebleeding (which may necessitate either further angiography and/or surgery) and the risk of compromise to hepatic artery flow. Change in enzymatic LFTs in this series is seen in Figure 2. This study also demonstrates the high risk of occlusion of endovascular hepatic artery stents resulting in a risk of liver ischemia, hepatic abscess, or liver necrosis. To set these data in context, it should be noted that in a Korean series of 17 patients undergoing endovascular stent for hemorrhage from the hepatic artery, the patency rate was 70% at 12 months with a policy of poststent anticoagulation.^14^ Further, the overall incidence of postpancreatectomy hemorrhage seen in this series is noted to be greater than the rate of 3.3% reported in a systematic review of bleeding after this procedure.^22^ More comprehensive prospective data capture in the present series may account for this difference. In the series from Hamburg, of 43 patients subjected to angiography, 25 underwent interventional coiling with a success rate of 80% (n = 20).^11^

In a contemporary series from Indiana, 28 of 647 patients with necrotizing pancreatitis (4.3%) developed a visceral artery pseudoaneurysm.^23^ The artery most commonly involved was the splenic artery (36%), followed by the GDA (24%) and 25 (89%) were managed by angiographic embolization. Data from a Boston study of over 1000 patients undergoing pancreatoduodenectomy indicate that postoperative bleeding can be an important cause of early readmission.^24^

Should patients receive routine antiplatelet therapy after endovascular hepatic artery stent placement? There is little direct evidence to guide an answer here. Although antiplatelet therapy is standard after the placement of coronary artery stents,^25^ in that setting, they are not being placed against a backdrop of major hemorrhage in a critically ill patient who has recently undergone major gastrointestinal surgery.

**FIGURE 3.**
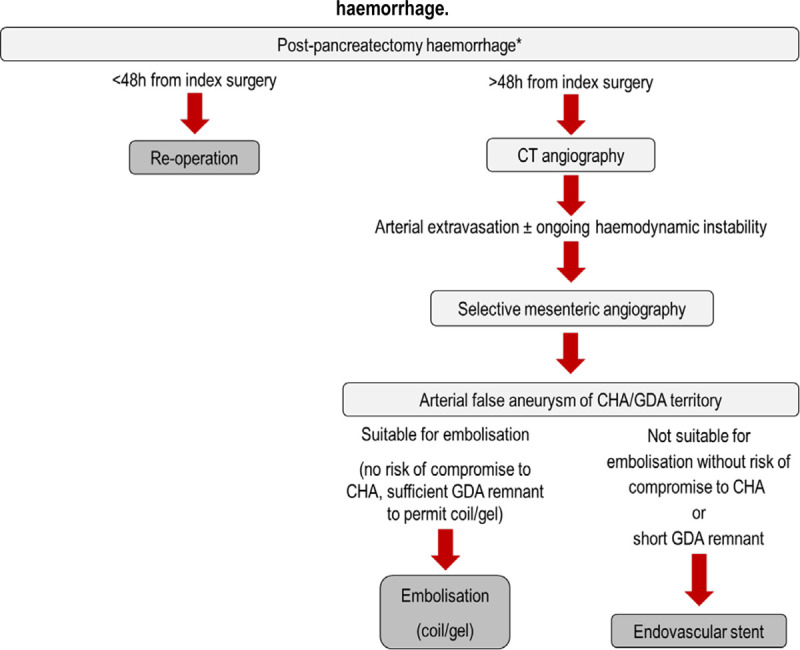
Algorithm for the use of hepatic artery endovascular stents in postpancreatectomy hemorrhage. Postpancreatectomy hemorrhage is defined by the criteria of the ISGPS.^15^ *Fiber-optic endoscopy for diagnosis and hemostasis can be considered in both early and late settings when there is evidence of gastrointestinal luminal hemorrhage. At all decision points in this algorithm, reoperation must be considered if the patient is insufficiently stable to sustain angiographic diagnosis or intervention. Arterial false aneurysm = pseudoaneurysm. CHA indicates common hepatic artery.

In summary, this series of 440 patients undergoing pancreatoduodenectomy in a single center has shown that 67 (15%) experienced postoperative hemorrhage. The data shown in this report can be used to construct a suggested contemporary management algorithm (Fig. 3). According to this, early postoperative hemorrhage would be managed by reoperation. It is accepted that early postoperative hemorrhage refers to the first 24 to 48 hours after surgery, following which there is a transition zone. Late postoperative hemorrhage can be investigated by CT angiography with selective mesenteric angiography then being used in those patients where extravasation has been demonstrated (or in those who have evidence of ongoing bleeding). Where feasible, coil or liquid embolization remains a first choice. However, in the specific settings of postpancreatoduodenectomy hemorrhage and either a short remnant GDA and/or direct bleed from the hepatic artery, embolization may not be feasible and, in this scenario, endovascular hepatic artery stent can be considered. The high risk of stent occlusion means that at the present time, hepatic endovascular stents for postpancreatectomy hemorrhage are likely regarded as treatments of last resort.

In conclusion, serious postpancreatoduodenectomy hemorrhage is a feared and potentially lethal complication. This study presents a modern management algorithm for this challenging complication and, for the first time, integrates the role of endovascular hepatic artery stents into the modern management of these patients.

**TABLE 2.** Details of Patients Undergoing Endovascular Stent Placement for Postpancreatectomy Hemorrhage (n = 7)

Patient	Anatomical Location	Stent	Cessation of Bleeding	Poststent Transfusion	Rebleeding	Further Intervention	Hepatic Infarction/Abscess
1	GDA stump pseudoaneurysm	6 × 22 mm E-ventus	Yes	Yes	No	No	No
2	GDA stump	7 × 37 mm E-ventus	No	Yes	Yes	Right adrenal pseudoaneurysm embolization	Abscess
3	GDA stump pseudoaneurysms	5 × 22 mm E-ventus	Yes	Yes	No	No	Infarction
4	Proximal common hepatic artery pseudoaneurysm	5 × 24 mm Bentley	Yes	No	No	No	Abscess
5	No clear evidence of bleed	5 × 50 mm Viabahn	No	Yes	Yes	No	Abscess
6	Proximal common hepatic artery pseudoaneurysm	7 × 23 mm Bentley	Yes	No	No	No	No
7	Distal common hepatic artery into hepatic artery proper	5 × 25 mm and 5 × 80 mm Bentley	Yes	Yes	Yes	Reoperation and common hepatic artery embolization	No

## ACKNOWLEDGMENTS

The authors acknowledge our former colleagues, Dr Nicholas Chalmers, Dr Finn Farquharson, and Dr Ray Ashleigh, the medical and nursing staff of the Hepatobiliary Unit of the Manchester Royal Infirmary and Sister Tehseen Khan.
